# Association of Prodromal Parkinson's Disease‐Like Features in Long COVID With Dream‐Enactment Behaviours

**DOI:** 10.1111/jsr.70371

**Published:** 2026-05-31

**Authors:** Siyi Gong, Yaping Liu, Bei Huang, Ngan Yin Chan, Eemil Partinen, Christian Benedict, Bjørn Bjorvatn, Ilona Merikanto, Luigi De Gennaro, Yves Dauvilliers, Brigitte Holzinger, Juliana Yordanova, Maria Korman, Cátia Reis, Anne‐Marie Landtblom, Sergio Mota‐Rolim, Michael R. Nadorff, Frances Chung, Yuichi Inoue, Harald Hrubos‐Strøm, Kentaro Matsui, Courtney J. Bolstad, Pei Xue, Colin A. Espie, Charles M. Morin, Thomas Penzel, Giuseppe Plazzi, Markku Partinen, Yun Kwok Wing

**Affiliations:** ^1^ Li Chiu Kong Family Sleep Assessment Unit, Department of Psychiatry, Faculty of Medicine The Chinese University of Hong Kong Hong Kong SAR China; ^2^ Centre for Sleep and Circadian Medicine The Affiliated Brain Hospital of Guangzhou Medical University Guangzhou Guangdong China; ^3^ Li Ka Shing Institute of Health Sciences, Faculty of Medicine The Chinese University of Hong Kong Hong Kong SAR China; ^4^ Department of Psychiatry Helsinki University Central Hospital Helsinki Finland; ^5^ Helsinki Sleep Clinic Terveystalo Healthcare Helsinki Finland; ^6^ Department of Clinical Neurosciences University of Helsinki, Clinicum Helsinki Finland; ^7^ Department of Pharmaceutical Biosciences Uppsala University Uppsala Sweden; ^8^ Department of Global Public Health and Primary Care University of Bergen, Norway and Norwegian Competence Centre for Sleep Disorders Bergen Norway; ^9^ Sleep Well Research Program, Faculty of Medicine University of Helsinki Helsinki Finland; ^10^ Orton Orthopedics Hospital Helsinki Finland; ^11^ Department of Psychology University of Rome Rome Italy; ^12^ Sleep‐Wake Disorders Unit, Department of Neurology Gui‐de‐Chauliac Hospital, CHU Montpellier, INM, INSERM, University of Montpellier Montpellier France; ^13^ Medical Universit Vienna, Postgraduate; Schlafcoaching; Institute für Consciousness and Dream Research Schlafcoaching Vienna Austria; ^14^ Institute of Neurobiology Bulgarian Academy of Sciences Sofia Bulgaria; ^15^ Department of Occupational Therapy, Faculty of Health Sciences Ariel University Ariel Israel; ^16^ Faculdade de Ciências Humanas Universidade Católica Lisbon Portugal; ^17^ Instituto de Medicina Molecular João Lobo Antunes Lisbon Portugal; ^18^ Faculdade de Medicina Universidade de Lisboa Lisbon Portugal; ^19^ Department of Medical Sciences Uppsala University Uppsala Sweden; ^20^ Department of Biomedical and Clinical Sciences Linköping University Linköping Sweden; ^21^ Brain Institute Federal University of Rio Grande Do Norte Natal Brazil; ^22^ Department of Psychology Mississippi State University Starkville Mississippi USA; ^23^ Department of Anesthesiology and Pain Medicine University Health Network, University of Toronto Toronto Canada; ^24^ Department of Somnology Tokyo Medical University Tokyo Japan; ^25^ Japan Somnology Center Institute of Neuropsychiatry Tokyo Japan; ^26^ Department of Otorhinolaryngology, Surgical Division Akershus University Hospital Oslo Norway; ^27^ Institute of Clinical Medicine University of Oslo, Campus Akershus University Hospital, Clinic of Surgery Lørenskog Norway; ^28^ Department of Clinical Laboratory National Centre Hospital, National Centre of Neurology and Psychiatry Tokyo Japan; ^29^ Birmingham VA Health Care System Birmingham Alabama USA; ^30^ Sir Jules Thorn Sleep & Circadian Neuroscience Institute (SCNi), Nuffield Department of Clinical Neurosciences University of Oxford Oxford England; ^31^ Centre de Recherche CERVO/Brain Research Center 'Ecole de Psychologie, Universit' e Laval Quebec City Canada; ^32^ Sleep Medicine Center Charite University Hospital Berlin Berlin Germany; ^33^ IRCCS–Istituto Delle Scienze Neurologiche di Bologna Bologna Italy; ^34^ Department of Biomedical, Metabolic and Neural Sciences University of Modena and Reggio Emilia Modena Italy

**Keywords:** coronavirus disease 2019 (COVID‐19), dream enactment behaviours (DEBs), international COVID‐19 sleep study (ICOSS), long COVID, neurodegeneration, prodromal Parkinson's disease (PD)

## Abstract

Emerging evidence links COVID‐19 to the predisposition of Parkinson's disease (PD). However, the relationship between long COVID and prodromal PD‐like features remains unclear, particularly in long COVID participants with dream‐enactment behaviours (DEBs) that may be suggestive of possible REM sleep behaviour disorder. This study aimed to quantify the burden of prodromal PD‐like features in long COVID. This online survey (May–Nov 2021) across 16 countries/regions included 11,261 participants. Data on demographics, COVID‐19 diagnosis, long COVID symptoms, sleep features and other typical prodromal PD‐like features were collected. The likelihood ratio (LR) of prodromal PD was calculated as a proxy for each participant's overall burden of prodromal PD‐like features, based on the 2019 Movement Disorder Society research criteria. Participants with long COVID (*n* = 1155) exhibited more symptoms suggestive of prodromal PD‐like features, including DEBs, olfactory dysfunctions, constipation, excessive daytime sleepiness, postural dizziness, depression with/without anxiety, urinary dysfunctions, cognitive impairment and a higher LR of prodromal PD when compared to non‐COVID‐19 participants and COVID‐19 recoverees. Long COVID was associated with a 73% higher burden of potential prodromal PD‐like features (adjusted odds ratio [aOR] = 1.73, 95% confidence interval [CI] = 1.57–1.90). Among those with long COVID, emergence or exacerbation of post‐infection DEBs further increased this burden by 38% (aOR = 1.38, 95% CI = 1.19–1.60). Our study suggested that long COVID is associated with an increased burden of prodromal PD‐like features, which appears to be further enhanced with DEBs.

## 1 Introduction

Coronavirus Disease 2019 (COVID‐19), which is caused by Severe Acute Respiratory Syndrome Coronavirus 2 (SARS‐CoV‐2), was a worldwide pandemic with more than 770 million confirmed cases and 7.1 million deaths up to Mar 2025 (WHO n.d.). Increasing evidence suggests that a series of long‐term complications may occur in individuals contracting COVID‐19, known as long COVID (Soriano et al. 2022) at which fatigue, shortness of breath, cognitive complaints, and respiratory, cardiac, and neurological symptoms are commonly reported (Soriano et al. 2022; *Nature Medicine* 2022; Groff et al. 2021).

Prior findings have raised the possibility of predisposition of patients with COVID‐19 to the risk of Parkinson's disease (PD). Historically, H1N1 pandemic (i.e., Spanish flu) was linked to the occurrence of ‘encephalitis lethargica’ and post‐encephalic Parkinsonism (Leta et al. 2022). Emerging evidence from recent case reports/series suggested the new onset of Parkinsonism with neuroimaging evidence among those with acute COVID‐19 (Cavallieri et al. 2022). A large cohort study (*n* = 236,379) reported the incidences of Parkinsonism and dementia 6 months after COVID‐19 were 0.11% and 0.67%, respectively (Taquet et al. 2021). Nevertheless, there are limited studies assessing the effect of long COVID on the risk of PD. In particular, previous studies have reported worsening of motor function in PD patients with long COVID (Bougea et al. 2023; Leta et al. 2021), indicating that long COVID might be involved in the progression of PD. Additionally, prior findings suggested that patients with long COVID may experience more severe symptoms such as fatigue, depression, anxiety, cognitive impairment and excessive daytime sleepiness (EDS) (Merikanto et al. 2023; Chen et al. 2023; Chau et al. 2024), which are also common correlates of PD (Heinzel et al. 2019; Chaudhuri and Behan 2004). In this regard, further studies are warranted to investigate the effect of long COVID on the potential PD risk.

REM sleep behaviour disorder (RBD) is considered as the most specific prodrome of α‐synucleinopathies (Hogl et al. 2018), characterized by recurrent dream‐enactment behaviours (DEBs) and REM sleep without atonia (RSWA) documented in video‐polysomnography (v‐PSG) (Sixel‐Doring et al. 2014). DEBs include motor behaviours (e.g., thrashing and kicking), and vocalizations (e.g., talking and screaming) (Fernandez‐Arcos et al. 2016). Prior findings indicated that COVID‐19 might be linked to the onset of RBD features, as suggested by 2‐ to 3‐fold increase in DEBs prevalence (Liu et al. 2023) and elevated RWSA (Steele et al. 2022). However, the potential impact of RBD features, especially in the presence of long COVID, on the predisposition to the PD risk remains unclear. This study aimed to evaluate whether long COVID is associated with an increased burden of potential prodromal PD‐like features and whether the occurrence of DEBs in participants with long COVID may further enhance the potential burden.

## 2 Methods

### 2.1 Study Design

This cross‐sectional study, as part of the second International COVID‐19 Sleep Study (ICOSS‐II) (Bjorvatn et al. 2025), was conducted online between May and November 2021 in 16 countries/regions including Austria, Brazil, Bulgaria, Canada, Hong Kong (China), Croatia, Finland, France, Germany, Israel, Italy, Japan, Norway, Portugal, Sweden and the United States of America (USA). The survey was advertised across various platforms, including university websites, newspapers, television and social media like Facebook and Twitter. The survey includes a set of harmonized questionnaires for all participants, along with targeted conditional questions for individuals who reported a history of coronavirus infection, vaccination, or some particular sleep symptoms or disorders. Sleep, circadian rhythm, health and mental well‐being were evaluated separately regarding the time before and during the pandemic and were further assessed conditionally with respect to the time before and after coronavirus infection.

Participants aged 18 years or older were invited through the abovementioned platforms and completed the anonymous survey online via platforms including Redcap and Qualtrics. A total of 15,723 participants completed the study, providing their age, sex, nationality and informed consent. Altogether, 11,261 participants were included in the final sample if their DEB, COVID and long COVID status were available. A total of 3196 participants had missing DEB status, while COVID and long COVID status were non‐computable for 1027 and 239 participants, respectively. Non‐computable COVID status indicates a response of ‘I don't know’ to the question assessing COVID‐19 symptoms. Non‐computable long COVID status indicated either some or all responses to the questions assessing long COVID symptoms were missing (Figure 1).

**FIGURE 1:**
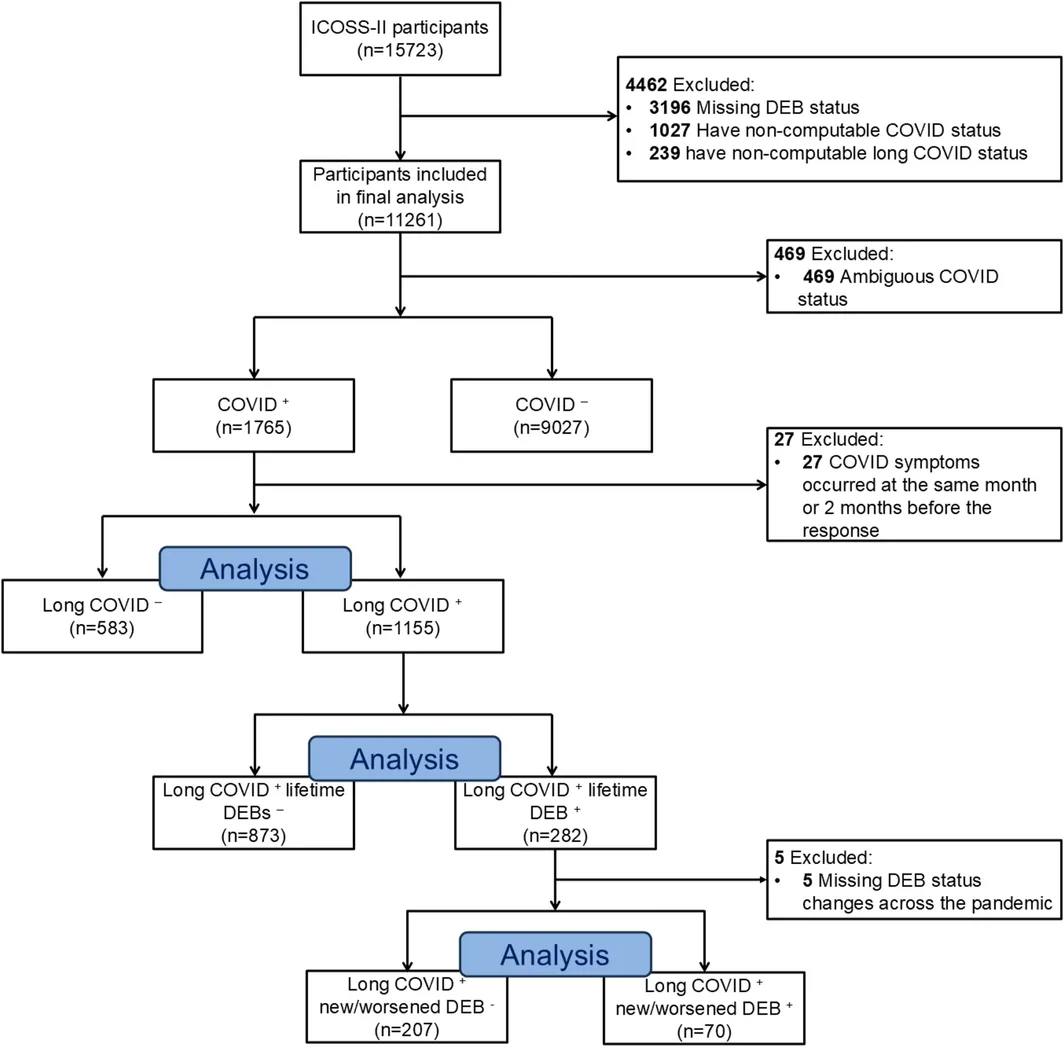
Flow chart for the inclusion of the participants.

### 2.2 Standard Protocol Approvals, Registrations and Patient Consents

The study protocol was compliant with the Declaration of Helsinki and followed the Strengthening the Reporting of Observational Studies in Epidemiology (STROBE) reporting guideline. Ethics approvals were obtained from each research ethics committee of the local institute in each participating country/region, except in Austria, Brazil, Finland, France, Norway and Sweden, where they were deemed unnecessary due to the survey's anonymous nature (Xue et al. 2024). Participants implicitly granted their consent by agreeing to participate in the study.

### 2.3 Measurement

The survey consisted of 47 questions and the estimated completion time for patients with COVID‐19 was about 40 min.

#### 2.3.1 Definitions of COVID and Long COVID

Participants were considered as COVID‐19 positive if they reported both having COVID‐19 symptoms (Q5a) and having been tested positive for COVID‐19 (Q5b). Conversely, participants were classified as COVID‐negative cases if they reported no symptoms of COVID‐19 and tested negative for COVID‐19. Furthermore, participants who reported either having COVID‐19 symptoms with a negative test result or a positive test result with no COVID‐19 symptoms were considered to have ambiguous COVID status and were further excluded (*n* = 469). Long COVID was defined as the presence of both confirmed COVID‐19 and any symptom (detailed in eAppendix 1 in Supporting Information) persisting for at least 3 months (Soriano et al. 2022). Participants whose symptoms occurred in the same month or 2 months before the response (i.e., first COVID symptoms occurred at least 3 months before the response to allow the development of long COVID) were excluded (*n* = 27).

#### 2.3.2 DEBs and RBD Definitions

Lifetime DEBs (suggestive of possible RBD) were assessed with the RBD Single‐Question Screen (RBD1Q)—‘Have you ever been told, or suspected yourself, that you seem to “act out your dreams” while asleep (for example, punching, flailing your arms in the air, making running movements, etc.)’? (Postuma et al. 2012). Besides RBD1Q, we also surveyed the chronological relationship between DEBs and COVID‐19 with the following question — ‘If you have had COVID‐19, how was the experience of “act out of dreams” related to the infection’? (response options: ‘less occurrences’, ‘no change’, ‘more occurrences’ or ‘occurred only after the infection’). The emergence or exacerbation of post‐infection DEBs was defined as a response of ‘more occurrences’ or ‘occurred only after the infection’ to the above question.

#### 2.3.3 Potential Confounders

We included potential confounders as follows: age, sex, pre‐existing medical conditions (sleep apnea), vaccination status, COVID‐19 to survey interval, mental factors (perceived stress level and post‐traumatic stress disorder (PTSD) symptoms) and socioeconomic features (ethnicity, marital status, financial burden and being living alone). Additionally, short sleepers were included due to the reported association with long COVID (Berezin et al. 2024). Short sleepers were defined as those whose average sleep duration is < 6 h per night. Pre‐existing medical conditions were defined as the diagnosed health conditions present before the pandemic, including sleep apnea (Q7). PTSD symptoms were evaluated with a two‐item self‐report measure (Lang et al. 2012). The perceived stress level was assessed with a single question: ‘Stress is a condition in which a person feels tense, troubled, nervous or anxious or sleep is troubled because of things bothering in mind. Do you feel such stress at the moment’? Respondents indicated their level of stress based on the extent of self‐perceived stress (Elo et al. 2003).

#### 2.3.4 Potential Neurodegenerative Biomarkers and Likelihood Ratio (LR) of Prodromal PD

Given long prodromal period before the emergence of PD, the movement disorder society (MDS) has proposed a list of risk markers (e.g., male sex, regular pesticide exposure, diabetes mellitus (DM) and physical inactivity) and prodromal markers (e.g., constipation, depression, possible RBD and EDS) that may suggest the presence of the potential prodromal PD features (MDS‐2019) (Heinzel et al. 2019). Herein, we incorporated risk markers, including male sex, DM and physical inactivity, alongside prodromal markers like possible RBD, olfactory dysfunctions, constipation, EDS, postural dizziness, urinary dysfunctions, depression with/without anxiety and cognitive impairment. Definitions for these markers are provided below.

EDS was assessed with the Epworth Sleepiness Scale (ESS) (Johns 1991; Chen et al. 2002) and the following question — ‘Do you feel excessively sleepy during daytime currently’? (Sarkanen et al. 2023). EDS was defined by either the ESS score equal to or larger than 11 or by answering at least 3 days per week to the above question (Johns 1991; Sarkanen et al. 2023). Constipation was identified as bowel movement less than three times per week. Olfactory dysfunctions were defined as a response of ‘somewhat worse’ or ‘much worse’ to the following question—‘Has your sense of smell (olfactory sense) changed during COVID‐19 compared to the time before you had COVID‐19’? Postural dizziness, urinary problems and cognitive symptoms (inattention and/or brain fog, and memory problems) were considered as symptoms suggestive of orthostatic hypotension, urinary dysfunctions and cognitive impairment, respectively.

The LR of prodromal PD was estimated by incorporating risk markers and prodromal markers as based on the MDS 2019 research criteria for prodromal PD (Heinzel et al. 2019). If the risk factors or biomarkers were not assessed in this study, we assigned a value of 1 for them when calculating the LR (detailed in eAppendix 2 in Supporting Information). Probable and possible prodromal PD were defined when the probability of prodromal PD was > 80% and 30%–80%, respectively (Heinzel et al. 2019).

### 2.4 Statistical Analysis

Normality tests were verified using the Shapiro–Wilk normality test. Categorical variables were reported as number and percentage (%), whereas continuous data were presented as mean with standard deviation (SD) for normally distributed data, and as median with interquartile range (IQR) for non‐normally distributed data. To correct the under/oversampling of certain subpopulations, the data were weighted by the post‐stratification weighting method based on crowd‐scouring. Specifically, weights for age and sex were calculated based on the population distribution of age and sex in each country/region (United Nations 2021) and then applied to each participant for post‐stratification adjustment.

For the demographic and clinical variables, Mann–Whitney *U*‐tests or independent sample *t*‐tests were used to analyse quantitative data, and categorical data were compared with chi‐square tests. For the prodromal PD markers and related LRs, Mann–Whitney *U*‐tests, Kruskal–Wallis or chi‐square tests were applied when appropriate. Multiple comparisons were performed using Bonferroni method to control the false positive rate at > 5%, which is considered as statistically significant. Logistic regression analyses were performed to examine the impact of long COVID on markers of prodromal PD after adjustment for age and sex. In particular, a generalized linear model (GLM) was applied to assess the impact of long COVID on potential prodromal PD‐like feature (total estimated LR with log‐transformed) after adjustment for confounders.

To ensure the robustness of our results, we conducted further sensitivity analyses by excluding six items from long COVID defining questions, including EDS, postural dizziness, urinary dysfunction, cognitive impairment (g1 and g2) and olfactory dysfunctions (eAppendix 2 in Supporting Information). These symptoms were removed to avoid potential confounding as they might overlap with prodromal PD‐like features. Additionally, to address the potential selection bias arising from the exclusion of participants with positive SARS‐CoV‐2 test while being asymptomatic (*n* = 33), we included participants with a positive test regardless of their symptom status. Forest plots for the markers of potential prodromal PD‐like features with statistical significance were plotted using R package ‘ggplot2’ (version 3.5.1). All the statistical analyses were performed with IBM SPSS Statistics, version 27.0 (IBM Corporation, Armonk, NY, USA). A two‐tailed *p* < 0.05 was regarded as statistically significant.

## 3 Results

### 3.1 Demographic, Socioeconomic and Clinical Characteristics

A total of 11,261 participants were included in the final analysis, 1765 participants were COVID‐positive cases, whereas 9027 participants were COVID‐negative cases after excluding 469 participants with ambiguous COVID status. Among the 1765 COVID‐positive participants, 65.4% of the participants with COVID‐19 (*n* = 1155) reported long COVID, while 583 (33.0%) participants were categorized as negative for long COVID (COVID‐19 recoverees) (Figure 1).

Compared with COVID‐19 recoverees, participants with long COVID were generally older (mean age: 48.6 ± 16.3 vs. 38.7 ± 17.9, *p* < 0.001), more often female (59.9% vs. 41.3%, *p* < 0.001), attained a lower educational level (62.3% vs. 68.9%, *p* = 0.006), and had a higher proportion receiving vaccination, though mostly limited to one vaccination (36.6% vs. 22.8%, *p* < 0.001). Concerning socioeconomic characteristics, participants with long COVID were predominantly Caucasian (80.1%), married (66.1%) and regularly employed (44.0%) (Table 1). Participants with long COVID had more lifetime DEBs (24.3% vs. 15.9%, *p* < 0.001), and emergence/exacerbation of post‐infection DEBs than COVID‐19 recoverees (10.9% vs. 7.5%, *p* = 0.015) (Table 1).

**TABLE 1:** Demographic, clinical, socioeconomic and DEB characteristics of COVID‐19 recoverees and participants with long COVID (weighted sample).

	COVID‐19 recoverees (*n* = 685)	Participants with long COVID (*n* = 1162)	*p* ^a^	aOR (95% CI)	*p* ^b^
Demographical and clinical features					
Age, mean (SD), years	38.7 (17.9)	48.6 (16.3)	**< 0.001**	—	
Sex, female	283 (41.3)	696 (59.9)	**< 0.001**	—	
Education level (university)	441 (68.9)	654 (62.3)	**0.006**	NA	0.13
BMI, kg/m^2^	26.7 ± 8.7	27.0 ± 7.0	0.46	NA	0.57
Vaccination dosage					
No vaccination	273 (39.9)	350 (30.2)	**< 0.001**	1 [Reference]	
One vaccination	156 (22.8)	424 (36.6)	**< 0.001**	1.81 (1.41–2.34)	**< 0.001**
Two vaccinations	256 (37.4)	385 (33.2)	0.07	1.22 (0.96–1.55)	0.10
COVID‐19 to survey interval, months	8.1 ± 6.4	9.5 ± 4.5	**< 0.001**	1.06 (1.04–1.09)	**< 0.001**
Socioeconomics					
Ethnicity			**< 0.001**		
Caucasian	513 (77.0)	868 (80.1)		1 [Reference]	
Asian	85 (12.8)	95 (8.8)		0.39 (0.27–0.55)	**< 0.001**
Others	68 (10.2)	120 (11.1)		1.22 (0.87–1.72)	0.25
Marital status			**< 0.001**		
Married	322 (48.7)	716 (66.1)		1 [Reference]	
Single	321 (48.6)	245 (22.6)		0.51 (0.39–0.65)	**< 0.001**
Divorced/widowed	18 (2.7)	122 (11.3)		1.90 (1.11–3.24)	**0.02**
Work status			**< 0.001**		
Retired	55 (8.0)	190 (16.3)		1 [Reference]	
Regular work	277 (40.4)	512 (44.0)		NA	0.13
Irregular work/shift worker	77 (11.2)	197 (16.9)		NA	**0.03**
Unemployed	38 (5.5)	95 (8.2)		NA	0.50
Financial burden	252 (36.8)	462 (39.8)	0.26	1.41 (1.14–1.74)	**0.001**
Living alone	150 (22.6)	198 (18.4)	**< 0.001**	0.66 (0.51–0.85)	**0.001**
DEB features					
Lifetime DEBs	109 (15.9)	282 (24.3)	**< 0.001**	1.96 (1.52–2.53)	**< 0.001**
Emergence/exacerbation of post‐infection DEBs	45 (7.5)	113 (10.9)	**0.02**	NA	0.16

*Note:* Numbers shown in the table are weighted using post‐stratification weighting method and therefore may differ from original sample sizes reported in the flowchart.

Abbreviations: aOR, adjusted odds ratio; BMI, body mass index; CI, confidence interval; COVID, Coronavirus Disease; DEBs, dream‐enactment behaviours; NA, not available; SD, standard deviation.

^a^ *p* value was calculated with *t*‐test or chi‐square test (all two‐sided tests).

^b^ *p* value was calculated with logistic regression analyses with dependent variable being long COVID after adjustment for age and sex.

* indicates *p* < 0.05.

** indicates *p* < 0.01.

*** indicates *p* < 0.001 (in bold).

### 3.2 Burden of Potential Prodromal PD‐Like Features in Participants With Long COVID

Participants with long COVID had a significantly higher proportion of the following prodromal markers (*p* < 0.001): DEBs (possible RBD), olfactory dysfunction, constipation, EDS, postural dizziness, urinary dysfunction, depression with/without anxiety, cognitive impairment and hence, a higher LR of prodromal PD (*p* < 0.001) when compared with non‐COVID‐19 participants and COVID‐19 recoverees (Table 2, Figure 2). After further adjustment for age, sex, short sleepers, pre‐existing medical conditions, perceived stress level, PTSD symptoms, ethnicity, marital status, living alone, financial burden, COVID‐19 to survey interval, and vaccination status, long COVID was associated with a 73% higher burden of potential prodromal PD‐like features (adjusted odds ratio [aOR] = 1.73, 95% CI = 1.57–1.90, *p* < 0.001, Table 2).

**TABLE 2:** MDS likelihood ratio of prodromal PD among ICOSS‐II non‐COVID‐19 participants, COVID‐19 recoverees and participants with long COVID (weighted sample).

	Participants without COVID‐19^1^ (*n* = 8738)	COVID‐19 recoverees^2^ (*n* = 685)	Participants with long COVID^3^ (*n* = 1162)	*p* ^a^	Post hoc analysis^b^	Unadjusted OR, 95% CI	aOR, 95% CI	*p* ^**c**^
Age, mean (SD), years	50.0 (18.2)	38.7 (17.9)	48.6 (16.3)	**< 0.001**	2 < 3 < 1	NA	NA	NA
Risk factors								
Sex, male	4463 (51.1)	402 (58.7)	466 (40.1)	**< 0.001**	3 < 1 < 2	NA	NA	NA
DM	471 (5.4)	20 (2.9)	72 (6.2)	**0.007**	2 < 1 = 3	NA	NA	0.15
Physical inactivity	4460 (56.2)	304 (51.4)	555 (58.2)	**0.03**	2 < 3	1.32 (1.07–1.62)**	NA	0.22
Prodromal markers								
Possible RBD	1817 (20.8)	109 (15.9)	282 (24.3)	**< 0.001**	2 < 1 < 3	1.69 (1.33–2.16)***	2.06 (1.59–2.67)	**< 0.001**
Olfactory dysfunctions	NA	274 (42.3)	594 (56.0)	**< 0.001**	2 < 3	1.73 (1.42–2.11)***	2.06 (1.67–2.54)	**< 0.001**
Constipation	2840 (32.8)	233 (34.3)	514 (44.5)	**< 0.001**	1 = 2 < 3	1.54 (1.26–1.87)***	1.29 (1.05–1.58)	**0.017**
EDS	2891 (35.0)	247 (37.7)	644 (57.9)	**< 0.001**	1 = 2 < 3	2.27 (1.86–2.76)***	2.39 (1.95–2.93)	**< 0.001**
Postural dizziness	1334 (15.3)	142 (20.7)	453 (39.1)	**< 0.001**	1 < 2 < 3	2.45 (1.97–3.05)***	2.78 (2.20–3.52)	**< 0.001**
Urinary dysfunctions	691 (7.9)	32 (4.7)	163 (14.1)	**< 0.001**	2 < 1 < 3	3.36 (2.27–4.97)***	2.91 (1.95–4.35)	**< 0.001**
Depression (± anxiety)	1353 (15.5)	83 (12.1)	305 (26.4)	**< 0.001**	1 = 2 < 3	2.58 (1.98–3.35)***	2.40 (1.84–3.13)	**< 0.001**
Cognitive impairment	981 (11.3)	71 (10.4)	541 (47.0)	**< 0.001**	1 = 2 < 3	7.67 (5.85–10.10)***	7.05 (5.36–9.27)	**< 0.001**
MDS likelihood ratio of prodromal PD								
Total estimated LR with log‐transformed, median (IQR)	0.3 (0.2–0.7)	0.4 (0.2–1.0)	0.9 (0.4–1.7)	**< 0.001**	1 < 2 < 3	1.71 (1.56–1.88)***	1.73 (1.57–1.90)	**< 0.001** ^**d**^
Possible prodromal PD	311 (3.6)	14 (2.0)	178 (15.3)	**< 0.001**	1 = 2 < 3	8.47 (4.90–14.62)***	8.47 (4.90–14.62)	**< 0.001**
Probable prodromal PD	11 (0.1)	7 (1.0)	35 (3.0)	**< 0.001**	1 < 2 < 3	2.93 (1.31–6.58)**	NA	0.14

*Note:* Numbers shown in the table are weighted using post‐stratification weighting method and therefore may differ from original sample sizes reported in the flowchart. Superscipt numbers refers to the three groups.

Abbreviations: aOR, adjusted odds ratio; CI, confidence interval; COVID, Coronavirus Disease; DEBs, dream‐enactment behaviours; DM, diabetes mellitus; EDS, excessive daytime sleepiness; ICOSS, The International Coronavirus Disease 2019 Sleep Study; IQR, interquartile range; LR, likelihood ratio; MDS, movement disorder society; OH, orthostatic hypotension; PD, Parkinson's disease; RBD, rapid eye movement sleep behaviour disorder; SD, standard deviation.

^a^ *p* value was calculated with One‐way ANOVA, Kruskal–Wallis test or chi‐square test (all two‐sided tests).

^b^ Bonferroni method was used to adjust *p* values generated by multiple comparisons, a false positive rate less than 5% (*q* value < 0.05) was accepted and indicated statistical significance.

^c^ *p* value was calculated with logistic regression analyses with dependent variable being long COVID after adjustment for age and sex.

^d^ *p* value was calculated with a generalized linear model with dependent variable being the total estimated LR with log transformation after adjustment for age, sex, short sleepers, pre‐existing medical conditions, perceived stress level, post‐traumatic stress disorder symptoms, ethnicity, marital status, living alone, financial burden, COVID‐19 to survey interval and vaccination status.

* Indicates *p* < 0.05.

** Indicates *p* < 0.01.

*** Indicates *p* < 0.001 (in bold).

**FIGURE 2:**
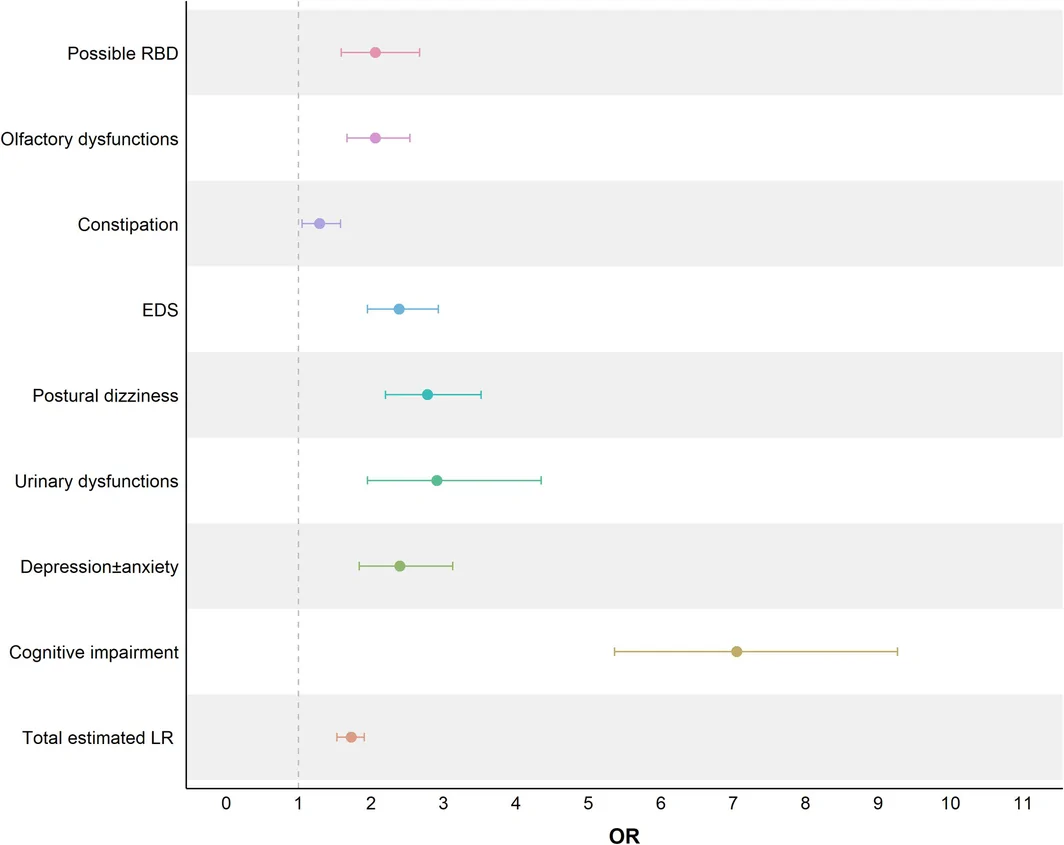
Forest plot of adjusted odds ratios for potential prodromal PD‐like features in participants with long COVID (weighted sample).

In the sensitivity analyses excluding six items from long COVID defining questions that may deem to overlap much with prodromal PD‐like features, similar results were observed. Participants with long COVID exhibited a higher level of all prodromal markers except olfactory dysfunctions and a higher LR of prodromal PD (*p* < 0.001) when compared with non‐COVID‐19 participants and COVID‐19 recoverees. After adjustment for abovementioned confounders, long COVID was associated with a 66% higher burden of potential prodromal PD‐like features (aOR = 1.66, 95% CI = 1.52–1.81, *p* < 0.001, Table 3). Additional sensitivity analyses including participants with a positive test regardless of their symptom status remained robust, and results showed that long COVID was associated with a 75% higher burden of potential prodromal PD‐like features (aOR = 1.75, 95% CI = 1.59–1.92, *p* < 0.001, eTable 3).

**TABLE 3:** MDS likelihood ratio of prodromal PD between participants with and without long COVID after excluding six overlapping prodromal PD features when defining long COVID (weighted sample).

	Participants without COVID‐19^1^ (*n* = 8738)	COVID‐19 recoverees^2^ (*n* = 807)	Participants with long COVID^3^ (*n* = 1038)	*p* ^a^	Post hoc analysis^b^	aOR, 95% CI	*p* ^**c**^
Age, mean (SD), years	50.0 (18.2)	39.0 (17.7)	49.5 (16.0)	**< 0.001**	2 < 1 = 3	—	—
Risk factors							
Sex, male	4463 (51.1)	462 (57.3)	405 (39.0)	**< 0.001**	3 < 1 < 2	—	—
DM	471 (5.4)	23 (2.9)	70 (6.8)	**0.001**	2 < 1 = 3	1.06 (1.04–1.07)	**< 0.001**
Physical inactivity	4460 (56.2)	350 (51.3)	510 (59.2)	**0.008**	2 < 1 = 3	NA	0.13
Prodromal markers							
Possible RBD	1817 (20.8)	126 (15.6)	265 (25.5)	**< 0.001**	2 < 1 < 3	2.35 (1.82–3.03)	**< 0.001**
Olfactory dysfunctions	NA	367 (48.4)	499 (52.6)	0.08	NA	1.36 (1.11–1.67)	**0.003**
Constipation	2840 (32.8)	263 (32.8)	484 (47.0)	**< 0.001**	1 = 2 < 3	1.55 (1.26–1.89)	**< 0.001**
EDS	2891 (35.0)	292 (38.0)	597 (59.8)	**< 0.001**	1 = 2 < 3	2.57 (2.11–3.14)	**< 0.001**
Postural dizziness	1334 (15.3)	165 (20.5)	431 (41.7)	**< 0.001**	1 < 2 < 3	3.35 (2.66–4.23)	**< 0.001**
Urinary dysfunctions	691 (7.9)	37 (4.6)	157 (15.2)	**< 0.001**	2 < 1 < 3	3.26 (2.23–4.76)	**< 0.001**
Depression (± anxiety)	1353 (15.5)	96 (12.0)	292 (28.2)	**< 0.001**	2 < 1 < 3	2.69 (2.08–3.47)	**< 0.001**
Cognitive impairment	981 (11.3)	109 (13.5)	501 (48.7)	**< 0.001**	1 = 2 < 3	5.59 (4.41–7.10)	**< 0.001**
MDS likelihood ratio of prodromal PD							
Total estimated LR with log‐transformed	0.3 (0.2–0.7)	0.5 (0.2–1.0)	1.0 (0.4–1.7)	**< 0.001**	1 < 2 < 3	1.66 (1.52–1.81)	**< 0.001** ^d^
Possible prodromal PD	311 (3.6)	18 (2.2)	175 (16.9)	**< 0.001**	1 = 2 < 3	8.70 (5.33–14.22)	**< 0.001**
Probable prodromal PD	11 (0.1)	8 (1.0)	34 (3.3)	**< 0.001**	1 < 2 < 3	NA	**0.05**

*Note:* Numbers shown in the table are weighted using post‐stratification weighting method and therefore may differ from original sample sizes reported in the flowchart.

Abbreviations: aOR, adjusted odds ratio; CI, confidence interval; COVID, Coronavirus Disease; DM, diabetes mellitus; EDS, excessive daytime sleepiness; LR, likelihood ratio; MDS, Movement Disorder Society; OH, orthostatic hypotension; PD, Parkinson's disease; RBD, rapid eye movement sleep behaviour disorder; SD, standard deviation.

^a^ *p* value was calculated with One‐way ANOVA, Kruskal–Wallis test or chi‐square test (all two‐sided tests).

^b^ Bonferroni method was used to adjust *p* values generated by multiple comparisons, a false positive rate less than 5% (*q* value < 0.05) was accepted and indicated statistical significance.

^c^ *p* value was calculated with logistic regression analyses with the dependent variable being long COVID after adjustment for age and sex.

^d^ *p* value was calculated with a generalized linear model with dependent variable being the total estimated LR with log transformation after adjustment for age, sex, short sleepers, pre‐existing medical conditions, perceived stress level, post‐traumatic stress disorder symptoms, ethnicity, marital status, living alone, financial burden, COVID‐19 to survey interval and vaccination status.

* indicates *p* < 0.05.

** indicates *p* < 0.01.

*** indicates *p* < 0.001 (in bold).

### 3.3 Burden of Potential Prodromal PD‐Like Features in Long COVID Participants With DEBs

We further divided the long COVID group based on the presence of emergence/exacerbation of DEBs after COVID‐19. Long COVID participants with emergence or exacerbation of post‐infection DEBs exhibited more olfactory dysfunction (73.7% vs. 51.7%, *p* = 0.003), constipation (61.9% vs. 44.3%, *p* = 0.01), urinary dysfunction (22.6% vs. 12.3%, *p* = 0.05), cognitive impairment (73.0% vs. 45.9%, *p* < 0.001) and hence, a higher LR of prodromal PD (median: 1.9 vs. 1.4, *p* < 0.001) (Table 4, Figure 3). The difference in the total LR (log‐transformed) remained significant after excluding the possible RBD item when recalculating LR (median: 1.0 vs. 1.5, *p* < 0.001) (Table 4). When adjusting for age, sex, short sleepers, pre‐existing medical conditions, perceived stress level, PTSD symptoms, ethnicity, marital status, living alone, financial burden, COVID‐19 to survey interval and vaccination status, emergence or exacerbation of post‐infection DEBs was associated with a 38% higher burden of potential prodromal PD‐like features (aOR = 1.38, 95% CI = 1.19–1.60, *p* < 0.001, Table 4) in participants with long COVID.

**TABLE 4:** MDS likelihood ratio of prodromal PD between long COVID participants with and without emergence/exacerbation of DEBs after infection (weighted sample).

	Long COVID participants without emergence/exacerbation of DEBs after infection (*n* = 212)	Long COVID participants with emergence/exacerbation of DEBs after infection (*n* = 63)	*p* ^a^	aOR, 95% CI	*p* ^b^
Age, mean (SD), years	44.1 (16.3)	49.1 (15.2)	**0.03**	—	—
Risk factors					
Sex, male	111 (52.4)	26 (40.6)	0.10	—	—
DM	15 (7.1)	2 (3.2)	0.26	NA	0.17
Physical inactivity	88 (48.1)	32 (54.2)	0.41	NA	0.42
Prodromal markers					
Possible RBD	212 (100.0)	63 (100.0)	**NA**	NA	NA
Olfactory dysfunctions	104 (51.7)	42 (73.7)	**0.003**	2.59 (1.36–4.95)	**0.004**
Constipation	94 (44.3)	39 (61.9)	**0.01**	2.10 (1.18–3.73)	**0.012**
EDS	143 (70.1)	42 (67.7)	0.72	NA	0.74
Postural dizziness	89 (42.0)	35 (55.6)	0.06	2.01 (1.12–3.62)	**0.02**
Urinary dysfunction	26 (12.3)	14 (22.6)	**0.05**	NA	0.10
Depression (± anxiety)	74 (34.9)	26 (41.3)	0.36	NA	0.38
Cognitive impairment	95 (45.9)	46 (73.0)	**< 0.001**	3.20 (1.72–5.95)	**< 0.001**
MDS likelihood ratio of prodromal PD					
Total estimated LR with log‐transformed, median (IQR)	1.4 (0.8–1.9)	1.9 (1.2–2.6)	**< 0.001**	1.38 (1.19–1.60)	**< 0.001** ^**c**^
Possible prodromal PD	44 (20.7)	22 (34.9)	**0.02**	2.04 (1.10–3.78)	**0.02**
Probable prodromal PD	13 (6.1)	12 (19.0)	**0.002**	2.99 (1.25–7.14)	**0.01**
Total estimated LR with log‐transformed (exclude the possible RBD item), median (IQR)	1.0 (0.4–1.5)	1.5 (0.8–2.2)	**< 0.001**	1.46 (1.23–1.74)	**< 0.001** ^**c**^
Possible prodromal PD	22 (10.4)	20 (31.7)	**< 0.001**	4.00 (2.02–7.95)	**< 0.001**
Probable prodromal PD	6 (2.8)	3 (4.7)	0.44	NA	0.81

*Note:* Numbers shown in the table are weighted using post‐stratification weighting method and therefore may differ from original sample sizes reported in the flowchart.

Abbreviations: aOR, adjusted odds ratio; CI, confidence interval; COVID, Coronavirus Disease; DEBs, dream‐enactment behaviours; DM, diabetes mellitus; EDS, excessive daytime sleepiness; IQR, interquartile range.; LR, likelihood ratio; MDS, movement disorder society; OH, orthostatic hypotension; PD, Parkinson's disease; RBD, rapid eye movement sleep behaviourbehavior disorder; SD, standard deviation.

^a^ *p* value was calculated with *t*‐test, Mann–Whitney *U*‐test, or chi‐square test (all two‐sided tests).

^b^ *p* value was calculated with logistic regression analyses with the dependent variable being long COVID with DEBs after adjustment for age and sex.

^c^ *p* value was calculated with generalized linear model with dependent variable being the total estimated LR with log transformation after adjustment for age, sex, perceived stress level, post‐traumatic stress disorder symptoms, short sleepers, pre‐existing medical conditions, ethnicity, marital status, living alone, financial burden, COVID‐19 to survey interval and vaccination status.

* indicates *p* < 0.05.

** indicates *p* < 0.01.

*** indicates *p* < 0.001 (in bold).

**FIGURE 3:**
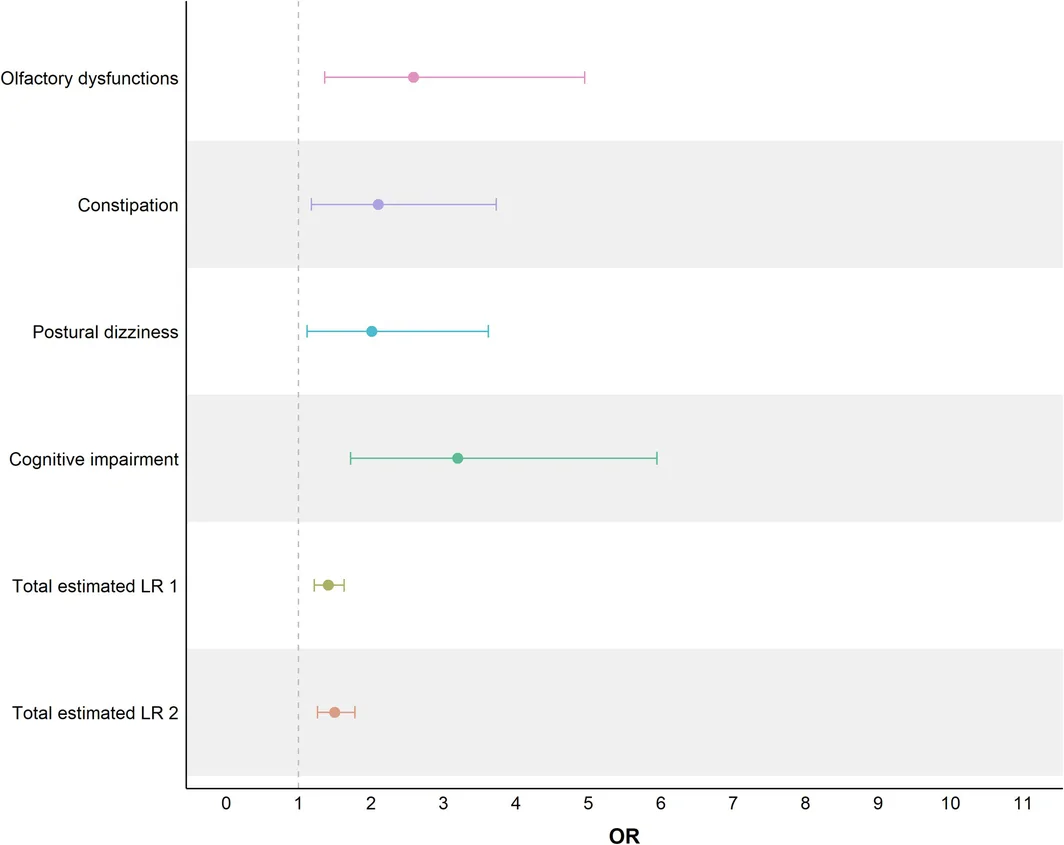
Forest plot of adjusted odds ratios for potential prodromal PD‐like features in long COVID participants with emergence/exacerbation of DEBs after infection.

Further sensitivity analyses demonstrated consistent results that long COVID participants with emergence/exacerbation of post‐infection DEBs were associated with a 39% higher burden of potential prodromal PD‐like features compared to those without (aOR = 1.39, 95% CI = 1.20–1.61, *p* < 0.001, eTable 1, eFigure 1). Additional sensitivity analyses including participants with a positive SARS‐CoV‐2 test regardless of symptom status showed similar results (eTable 4).

We also assessed the potential impact of lifetime DEBs in long COVID on neurodegenerative risk. Long COVID participants with lifetime DEBs had increased features such as constipation, EDS, postural dizziness, depression, cognitive impairment, and hence a higher LR of prodromal PD (median: 1.5 vs. 0.8, *p* < 0.001, eTable 2) when compared to those without. The difference in LR remained significant even after excluding the possible RBD item when recalculating LR (median: 1.0 vs. 0.8, *p* < 0.001, eTable 2, eFigure 2).

## 4 Discussion

This international study with 11,261 participants documented an association between long COVID and an elevated burden of potential prodromal PD‐like features, as reflected by a higher LR of prodromal PD. Additionally, the presence of DEBs (suggestive of possible RBD, a well‐characterized prodrome of PD), especially the emergence or exacerbation of post‐infection DEBs, further increased the potential burden of prodromal PD‐like features in participants with long COVID.

We identified an increased burden of potential prodromal PD‐like features in participants with long COVID. Given the overlap between long COVID symptoms and prodromal PD‐like features, we further conducted sensitivity analyses, and the burden remained after controlling for these overlaps. Previous research also suggested neuroinflammatory changes in patients with COVID‐19, which may predispose them to neurodegeneration. For instance, prior post‐mortem studies identified pronounced inflammatory changes in the brainstem and cerebellum in patients with COVID‐19 (Matschke et al. 2020). Additionally, a recent neuroimaging study reported long‐term microstructural brainstem abnormalities, consistent with the neuroinflammatory response, in patients with COVID‐19 (Rua et al. 2024). Thus, further studies are needed to determine the long‐term effects of COVID‐19 on the central nervous system (CNS).

We also found that the presence of DEBs further increased the burden of potential prodromal PD‐like features in participants with long COVID. Emergence/exacerbation of post‐infection DEBs seemed to be associated with a further elevated burden of prodromal PD‐like features. Nevertheless, DEBs symptoms may include cases of genuine idiopathic RBD (iRBD), isolated DEB and other false positives such as obstructive sleep apnea, non‐REM parasomnias and PTSD (Barone 2020; Yao et al. 2019; Wang et al. 2022). Although the association remained significant after adjustment for abovementioned potential confounders, this study lacked objective sleep measurements or clinical evaluations for these confounders. Thus, further studies are warranted to determine whether v‐PSG‐confirmed isolated DEB or iRBD will be exacerbated or precipitated by COVID‐19, especially among those with long COVID.

The specific mechanisms linking long COVID to potential neurodegenerative risk remain unclear. In brief, the deterioration of neurological functions following COVID‐19 may stem from direct neuroinvasion (e.g., via olfactory bulb or blood–brain barrier disruption) or/and an indirect systemic immune response (Huang et al. 2023). As for the direct invasion, prior studies suggested the virus may enter the CNS via olfactory bulb (Meinhardt et al. 2021), a pathway shared with viral pathogens in PD (Hawkes et al. 2007). Notably, olfactory decline was observed in both patients with long COVID (22%–64%) (Hu et al. 2023; Mendes Paranhos et al. 2022) and PD (75%) (Chen et al. 2015), serving as a prodromal marker of PD (Postuma et al. 2019). Our results also demonstrated more olfactory dysfunctions in participants with long COVID compared to those without, suggesting this shared olfactory pathway and a potential risk of prodromal PD. Additionally, preclinical in vitro studies suggested that SARS‐CoV‐2 infection may accelerate the α‐synuclein aggregation (Semerdzhiev et al. 2022) and induce Lewy body‐like pathology (Wu et al. 2022). However, as these are in vitro models, whether and how COVID‐19 will contribute to α‐synucleinopathy neurodegeneration in humans needs further investigations. As for the indirect immune response, systemic effects triggered by infection might play a role in subsequent neurodegeneration (Huang et al. 2023).

Furthermore, long COVID may be associated with potential neurodegenerative risk via shared clinical factors that concurrently serve as long COVID manifestations and prodromal PD features (Heinzel et al. 2019). These factors include behavioural changes (e.g., physical inactivity), mood disorders (e.g., anxiety, depression), dysautonomia (e.g., orthostatic hypotension) and circadian disturbances (Duret and Nagoshi 2025; Davis et al. 2023). Although we have excluded the long COVID question items that overlap with prodromal PD markers, there remains a possibility of symptom overlap between long COVID and neurodegenerative markers. In this regard, further studies are warranted to track the long‐term effects of long COVID on CNS.

There are several limitations of this present study. First, given the study is cross‐sectional and retrospective in nature, recall bias cannot be ruled out, and the use of a convenient sample limits the generalizability of the findings to the general population. Second, although we applied the post‐stratification weighting method, the response bias may not be entirely discounted as the online survey might be less accessible to the elderly or participants with more severe COVID‐19. Third, our proportion of long COVID (i.e., 65.4%) is comparable but at a relatively higher range compared to the overall prevalence of long COVID in the general population (i.e., 1.7%–90.4%) (Jeffrey et al. 2024; Ballering et al. 2022; Hastie et al. 2023; Wong et al. 2023), possibly due to the inconsistent long COVID definitions and our sampling strategy (i.e., specifically targeting long COVID populations in certain countries). Finally, the diagnosis of COVID‐19, DEBs history and proxies of prodromal PD features were assessed by self‐reported questionnaires without any clinical or v‐PSG confirmation (Steele et al. 2022). Reliance on subjective and potentially non‐specific symptoms, along with the absence of objective assessments and unspecified COVID‐19 testing modality (e.g., RT‐PCR, antigen rapid test, antibody/serology test), may have introduced misclassification bias. Future longitudinal studies with comprehensive, standardized COVID‐19 diagnostic testing, objective clinical evaluations (e.g., detailed assessment of cognitive deficits) and sleep assessments are needed.

## 5 Conclusion

In summary, this large‐scale multinational study identified an association between long COVID and an increased burden of potential prodromal PD‐like features. Moreover, the emergence or exacerbation of post‐infection DEBs further amplified the potential burden in long COVID. These findings underscore the importance of regular and long‐term evaluation of patients with long COVID, especially those with DEBs to monitor for subsequent potential neurodegeneration.

## Author Contributions

**Siyi Gong:** software, formal analysis, writing – original draft, writing – review and editing; **Yaping Liu:** data curation, project administration, supervision; **Bei Huang:** investigation, methodology; **Ngan Yin Chan:** investigation; **Eemil Partinen:** project administration, writing – review and editing; **Christian Benedict:** project administration, writing – review and editing; **Bjørn Bjorvatn:** project administration, writing – review and editing; **Ilona Merikanto:** project administration, writing – review and editing; **Luigi De Gennaro:** project administration, writing – review and editing; **Yves Dauvilliers:** project administration, writing – review and editing; **Brigitte Holzinger:** project administration, writing – review and editing; **Juliana Yordanova:** project administration, writing – review and editing; **Maria Korman:** project administration, writing – review and editing; **Cátia Reis:** project administration, writing – review and editing; **Anne‐Marie Landtblom:** project administration, writing – review and editing; **Sergio Mota‐Rolim:** project administration, writing – review and editing; **Michael R. Nadorff:** project administration, writing – review and editing; **Frances Chung:** project administration, writing – review and editing; **Yuichi Inoue:** project administration, writing – review and editing; **Harald Hrubos‐Strøm:** project administration, writing – review and editing; **Kentaro Matsui:** project administration, writing – review and editing; **Courtney J. Bolstad:** project administration, writing – review and editing; **Pei Xue:** project administration, writing – review and editing; **Colin A. Espie:** project administration, writing – review and editing; **Charles M. Morin:** project administration, writing – review and editing; **Thomas Penzel:** project administration, writing – review and editing; **Giuseppe Plazzi:** project administration, writing – review and editing; **Markku Partinen:** project administration, writing – review and editing; **Yun Kwok Wing:** conceptualization, project administration, writing – review and editing.

## Funding

The authors have nothing to report.

## Conflicts of Interest

The authors declare no conflicts of interest.

## Supporting information


**eAppendix 1** List of long COVID symptoms:
**eAppendix 2**. Overview of long COVID symptom questions and prodromal PD features.
**eTable 1**. MDS likelihood ratio of prodromal PD between long COVID participants with and without emergence/exacerbation of post‐infection DEBs after excluding six overlapping prodromal PD features when defining long COVID (weighted sample).
**eFigure 1**. Forest plot of adjusted odds ratios for potential prodromal PD‐like features in long COVID participants with and without emergence/exacerbation of post‐ infection DEBs after excluding six overlapping prodromal PD features when defining long COVID (weighted sample).
**eTable 2**. MDS likelihood ratio of prodromal PD between long COVID participants with and without lifetime DEBs (weighted sample).
**eFigure 2**. Forest plot of adjusted odds ratios for potential prodromal PD‐like features in long COVID participants with lifetime DEB (weighted sample).
**eTable 3**. MDS likelihood ratio of prodromal PD among ICOSS‐II non‐COVID‐19 participants, COVID‐19 recoverees and participants with long COVID for sensitivity analyses.
**eTable 4**. MDS likelihood ratio of prodromal PD between long COVID participants with and without emergence/exacerbation of DEBs after infection for sensitivity analyses.

## Data Availability

The data that support the findings of this study are available from the corresponding author upon reasonable request.
