# Supplementary material for: Association of Prodromal Parkinson's Disease‐Like Features in Long COVID With Dream‐Enactment Behaviours
Source: J Sleep Res. 2026 May 31;35(5):e70371. doi: 10.1111/jsr.70371 (PMC13619039; doi:10.1111/jsr.70371)
Supplement: Supplementary file 1 [file JSR-35-e70371-s001.docx]

# Supplemental content:

**eAppendix 1**. List of long COVID symptoms:

**eAppendix 2**. Overview of long COVID symptom questions and prodromal PD features.

**eTable 1**. MDS likelihood ratio of prodromal PD between long COVID participants with and without emergence/exacerbation of post-infection DEBs after excluding six overlapping prodromal PD features when defining long COVID (weighted sample).

**eFigure 1**. Forest plot of adjusted odds ratios for potential prodromal PD-like features in long COVID participants with and without emergence/exacerbation of post- infection DEBs after excluding six overlapping prodromal PD features when defining long COVID (weighted sample).

**eTable 2**. MDS likelihood ratio of prodromal PD between long COVID participants with and without lifetime DEBs (weighted sample).

**eFigure 2**. Forest plot of adjusted odds ratios for potential prodromal PD-like features in long COVID participants with lifetime DEB (weighted sample).

**eTable 3**. MDS likelihood ratio of prodromal PD among ICOSS-II non-COVID-19 participants, COVID-19 recoverees, and participants with long COVID for sensitivity analyses.

**eTable 4**. MDS likelihood ratio of prodromal PD between long COVID participants with and without emergence/exacerbation of DEBs after infection for sensitivity analyses.

# eAppendix 1. List of long COVID symptoms:

a) shortness of breath/difficulty breathing/chest pain, b) joint pain (arthralgia)/muscle pain, muscle aches, c) migraine, d) headache other than migraine, e) abdominal pains, colics, f) palpitations/cardiac arrhythmia, g) tachycardia, fast pulse rate, h) post exertional malaise referring to prolonged weakness/poor functionality after exertion, such as muscle weakness, difficulties walking long distances, i) dizziness when standing, j) low blood pressure (hypotension), k) urinary problems, l) problems of sweating/trouble tolerating cold/heat, m) problems of attention or concentration/brain fog, cognitive dysfunction, memory problems, n) loss of smell/taste, o) hallucinations, psychotic symptoms,

p) feverishness/flu-like symptoms, such as sore throat, runny nose, etc., q) diarrhea/nausea/vomiting, r) symptoms of fatigue, and s) excessive daytime sleepiness

# eAppendix 2. Overview of long COVID symptom questions and prodromal PD features.

| Long COVID series qx (Q8) | Prodromal PD features | | Corresponding item in survey questions |
| --- | --- | --- | --- |
| a. Fatigue | Risk markers | Male sex | Q3. gender |
| b. Shortness of breath or difficulty breathing and/or Chest pain |  | Regular pesticide exposure | NA |
| c. Joint pain (arthralgia) and/or muscle pain, muscle aches |  | Occupational solvent exposure | NA |
| d1. Migraine |  | Nonuse of caffeine | NA |
| D2. Headache, other than migraine |  | Nonsmoking  Current smoker  Never smoker  Former smoker | NA |
| D3. Abdominal pains, colics |  | First-degree relative with PD  or  Known gene mutation  or  Polygenic risk score | NA |
| e1. Excessive daytime sleepiness |  | SN hyperechogenicity | NA |
| e2. Difficulties falling or staying asleep |  | Diabetes mellitus (type II) | Q7_g. type 2 DM |
| f1. Palpitations and/or Cardiac arrhythmia |  | Physical inactivity | Q45. Exercise pattern |
| f2. Tachycardia, fast pulse rate |  | Low plasma urate levels | NA |
| f3. Post-exertional malaise referring to prolonged weakness/poor functionality after exertion, such as muscle weakness, difficulties walking long distances | Prodromal markers | PSG-proven RBD | NA |
| f4. Dizziness when standing |  | Possible RBD (questionnaire) | Q21a. RBD1Q |
| f5. Low blood pressure (hypotension) |  | Dopaminergic PET/SPECT clearly abnormal | NA |
| f6. Urinary problems |  | Subthreshold parkinsonism or  Abnormal quantitative motor testing | NA |
| f7. Problems of sweating and/or Trouble of tolerating cold/heat |  | Olfactory dysfunctions | Q47b. Olfactory decline |
| g1. Problems of attention or of concentration and/or Brain fog, cognitive dysfunction |  | Constipation | Q22. Constipation |
| g2. Memory problems |  | Excessive daytime somnolence | Q26 (EDS) or Q11a (“Do you feel excessively sleepy during daytime currently”) |
| g3. Loss of smell and/or taste |  | Orthostatic hypotension - neurogenic OH | NA |
| g4. Hallucinations, psychotic symptoms |  | Symptomatic OH | Q8_f4 |
| h1. Feverishness and/or flu-like symptoms, such as sore throat, runny nose etc. |  | Erectile dysfunction | NA |
| h2. Diarrhea and/or Nausea or vomiting |  | Urinary dysfunction | Q8_f6 |
|  |  | Depression (± anxiety) | Q7_w. depression |
|  |  | Global cognitive deficit | Q8_g1&g2 |

Abbreviations: COVID, Coronavirus Disease; PD, Parkinson’s disease; PSG, polysomnography; RBD, rapid eye movement sleep behavior disorder; OH, orthostatic hypotension; NA, not available.

PS: Items that are labeled as “NA” were assigned a value of 1 when calculating likelihood ratio of prodromal PD.

# eTable 1. MDS likelihood ratio of prodromal PD between long COVID participants with and without emergence/exacerbation of post-infection DEBs after excluding six overlapping prodromal PD features when defining long COVID (weighted sample).

|  | **Long COVID participants without emergence/exacerbation of DEBs after infection**  **(n = 196)** | **Long COVID participants with emergence/exacerbation of DEBs after infection**  **(n = 62)** | ***P* value^a^** | **aOR, 95% CI** | ***P* value^b^** |
| --- | --- | --- | --- | --- | --- |
| Age, mean (SD), y | 39.5 (14.0) | 45.2 (14.2) | **0.004** | - | - |
| ***Risk factors*** |  |  |  |  |  |
| Sex, male | 103 (52.6) | 26 (41.3) | 0.12 | - | - |
| DM | 13 (6.6) | 2 (3.2) | 0.53 | NA | 0.23 |
| Physical inactivity | 80 (48.2) | 32 (55.2) | 0.36 | NA | 0.54 |
| ***Prodromal markers*** |  |  |  |  |  |
| Possible RBD | 196 (100.0) | 62 (100.0) | - | - | - |
| Olfactory dysfunctions | 93 (50.5) | 42 (73.7) | **0.002** | 2.69 (1.40-5.17) | **0.003** |
| Constipation | 86 (44.1) | 39 (62.9) | **0.01** | 2.17 (1.21-3.90) | **0.01** |
| EDS | 136 (71.6) | 42 (68.9) | 0.68 | NA | 0.63 |
| Postural dizziness | 86 (43.9) | 35 (56.5) | 0.08 | NA | 0.06 |
| Urinary dysfunction | 25 (12.8) | 14 (23.0) | 0.06 | NA | 0.18 |
| Depression (±anxiety) | 70 (35.7) | 26 (41.3) | 0.43 | NA | 0.44 |
| Cognitive impairment | 87 (45.5) | 46 (74.2) | **<0.001** | 3.21 (1.66-6.18) | **0.001** |
| ***MDS likelihood ratio of prodromal PD*** |  |  |  |  |  |
| Total estimated LR with log-transformed | 1.5 (0.9-2.1) | 1.9 (1.2-2.7) | <0.001 | 1.39 (1.20-1.61) | **<0.001^c^** |
| Possible prodromal PD | 42 (21.4) | 22 (34.9) | 0.03 | 1.98 (1.06-3.68) | **0.03** |
| Probable prodromal PD | 13 (6.6) | 12 (19.0) | 0.004 | 3.00 (1.25-7.23) | **0.02** |
| Total estimated LR with log-transformed (exclude the possible RBD item) | 1.1 (0.5-1.7) | 1.5 (0.8-2.3) | <0.001 | 1.47 (1.23-1.76) | **<0.001^c^** |
| Possible prodromal PD | 29 (15.3) | 24 (34.8) | 0.001 | 3.83 (1.92-7.66) | **<0.001** |
| Probable prodromal PD | 2 (1.1) | 3 (4.3) | 0.12 | 1.12 (1.04-1.21) | **0.002** |

Abbreviations: MDS, movement disorder society; PD, Parkinson disease; DEBs, dream-enactment behaviors; COVID, Coronavirus Disease; aOR, adjusted odds ratio; CI, confidence interval; SD, standard deviation; DM, diabetes mellitus; RBD, rapid eye movement sleep behavior disorder; EDS, excessive daytime sleepiness; OH, orthostatic hypotension; LR, likelihood ratio.

Numbers shown in the table are weighed using post-stratification weighting method and therefore may differ from original sample sizes reported in the flowchart.

^a^ P value was calculated with t-test, Mann-Whitney U test or Chi square test, all two-sided tests.

^b^ P value was calculated with logistic regression analyses with dependent variable being long COVID with DEBs after adjustment for age and sex.

^c^ P value was calculated with generalized linear model with dependent variable being total estimated LR with log

transformation after adjustment for age, sex, perceived stress level, post-traumatic stress disorder symptoms, short sleepers, pre-existing medical conditions, ethnicity, marital status, living alone, financial burden, COVID-19 to survey interval, and vaccination status.

# eFigure 1. Forest plot of adjusted odds ratios for potential prodromal PD-like features in long COVID participants with and without emergence/exacerbation of post- infection DEBs after excluding six overlapping prodromal PD features when defining long COVID (weighted sample).

Abbreviations: PD, Parkinson disease; COVID, Coronavirus Disease; DEBs, dream-enactment behaviors; LR, likelihood ratio; Total estimated LR 1 = Total estimated LR with log-transformed; Total estimated LR 2 = Total estimated LR with log- transformed (exclude items of possible RBD).

# eTable 2. MDS likelihood ratio of prodromal PD between long COVID participants with and without lifetime DEBs (weighted sample).

|  | **Long COVID participants without lifetime DEBs**  **(n = 880)** | **Long COVID participants with lifetime DEBs**  **(n = 282)** | ***P* value^a^** | **aOR, 95% CI** | ***P* value^b^** |
| --- | --- | --- | --- | --- | --- |
| Age, mean (SD), y | 49.6 (16.3) | 45.3 (16.0) | **<0.001** | - | - |
| ***Risk factors*** |  |  |  |  |  |
| Sex, male | 325 (36.9) | 141 (50.0) | **<0.001** | - | - |
| DM | 54 (6.2) | 17 (6.0) | 0.93 | NA | 0.44 |
| Physical inactivity | 436 (61.7) | 120 (48.6) | **<0.001** | 0.61 (0.45-0.82) | **0.001** |
| ***Prodromal markers*** |  |  |  |  |  |
| Possible RBD | 0 (0.0) | 282 (100.0) | **<0.001** | NA | NA |
| Olfactory dysfunctions | 442 (55.5) | 152 (57.6) | 0.55 | NA | 0.89 |
| Constipation | 375 (42.9) | 139 (49.6) | **0.048** | 1.37 (1.05-1.80) | **0.02** |
| EDS | 452 (53.8) | 192 (70.3) | **<0.001** | 1.93 (1.44-2.60) | **<0.001** |
| Postural dizziness | 323 (36.9) | 130 (46.1) | **0.006** | 1.47 (1.11-1.95) | **0.007** |
| Urinary dysfunction | 121 (13.8) | 41 (14.6) | 0.73 | NA | 0.35 |
| Depression (± anxiety) | 204 (23.3) | 100 (35.5) | **<0.001** | 1.91 (1.43-2.57) | **<0.001** |
| Cognitive impairment | 393 (44.9) | 148 (53.4) | **0.01** | 1.61 (1.22-2.13) | **0.001** |
| ***MDS likelihood ratio of prodromal PD*** |  |  |  |  |  |
| Total estimated LR with log-transformed | 0.8 (0.3-1.4) | 1.5 (0.9-2.3) | <0.001 | 1.61 (1.48-1.75) | **<0.001^c^** |
| Possible prodromal PD | 107 (12.2) | 71 (25.2) | <0.001 | 2.42 (1.73-3.39) | **<0.001** |
| Probable prodromal PD | 10 (1.1) | 25 (8.9) | <0.001 | 12.94 (5.89-28.39) | **<0.001** |
| Total estimated LR with log- transformed (exclude possible RBD item) | 0.8 (0.3-2.1) | 1.0 (0.6-1.8) | <0.001 | 1.18 (1.07-1.30) | **0.01^c^** |
| Possible prodromal PD | 121 (13.7) | 48 (17.0) | 0.17 | NA | 0.16 |
| Probable prodromal PD | 10 (1.1) | 9 (3.2) | 0.03 | 4.86 (1.81-13.05) | **0.002** |

Abbreviations: MDS, movement disorder society; PD, Parkinson disease; DEBs, dream-enactment behaviors; COVID, Coronavirus Disease; aOR, adjusted odds ratio; CI, confidence interval; SD, standard deviation; DM, diabetes mellitus; RBD, rapid eye movement sleep behavior disorder; EDS, excessive daytime sleepiness; OH, orthostatic hypotension; LR, likelihood ratio.

Numbers shown in the table are weighed using post-stratification weighting method and therefore may differ from original sample sizes reported in the flowchart.

^a^ P value was calculated with t-test, Mann-Whitney U test or Chi square test, all two-sided tests.

^b^ P value was calculated with logistic regression analyses with dependent variable being long COVID with DEBs after adjustment for age and sex.

^c^ P value was calculated with generalized linear model with dependent variable being total estimated LR with log

transformation after adjustment for age, sex, perceived stress level, post-traumatic stress disorder symptoms, short sleepers, pre-existing medical conditions, ethnicity, marital status, living alone, financial burden, COVID-19 to survey interval, and vaccination status.

# eFigure 2. Forest plot of adjusted odds ratios for potential prodromal PD-like features in long COVID participants with lifetime DEB (weighted sample).

Abbreviations: PD, Parkinson disease; COVID, Coronavirus Disease; DEBs, dream-enactment behaviors; LR, likelihood ratio; EDS, excessive daytime sleepiness; LR, likelihood ratio.

Total estimated LR = Total estimated LR with log-transformed

**eTable 3. MDS likelihood ratio of prodromal PD among ICOSS-II non-COVID-19 participants, COVID-19 recoverees, and participants with long COVID for sensitivity analyses.**

|  | **Participants without COVID-19^1^**  **(n = 8738)** | **COVID-19 recoverees^2^**  **(n = 706)** | **Participants with long COVID^3^**  **(n = 1174)** | ***P* value^a^** | **Post-hoc analysis^b^** | **Unadjusted OR, 95% CI** | **aOR, 95% CI^c^** | ***P* value^c^** |
| --- | --- | --- | --- | --- | --- | --- | --- | --- |
| Total estimated LR with log-transformed, median (IQR) | 0.3 (0.2-0.7) | 0.4 (0.2-1.0) | 0.9 (0.4-1.6) | <0.001 | 1<2<3 | 1.73 (1.58-1.89) *** | 1.75 (1.59-1.92) | <0.001 |

^a^ P value was calculated with One-way ANOVA, Kruskal-Wallis test or Chi square test, all two-sided tests.

^b^ Bonferroni method was used to adjust p values generated by multiple comparisons, a false positive rate less than 5% (q value <0.05) was accepted and indicated statistical significance.

^c^ P value, aOR, and CI were calculated with generalized linear model with dependent variable being total estimated LR with log transformation after adjustment for age, sex, short sleepers, pre-existing medical conditions, perceived stress level, post-traumatic stress disorder symptoms, ethnicity, marital status, living alone, financial burden, COVID-19 to survey interval, and vaccination status.

* indicates *p* < 0.05, ** indicates *p* < 0.01, *** indicates *p* < 0.001.

**eTable 4. MDS likelihood ratio of prodromal PD between long COVID participants with and without emergence/exacerbation of DEBs after infection for sensitivity analyses**.

|  | **Long COVID participants without emergence/exacerbation of DEBs after infection**  **(n = 213)** | **Long COVID participants with emergence/exacerbation of DEBs after infection**  **(n = 63)** | ***P* value^a^** | **aOR, 95% CI^b^** | ***P* value^b^** |
| --- | --- | --- | --- | --- | --- |
| Total estimated LR with log-transformed, median (IQR) | 1.4 (0.8-1.9) | 1.9 (1.2-2.6) | <0.001 | 1.38 (1.19-1.60) | <0.001 |
| Total estimated LR with log-transformed, (exclude the possible RBD item), median (IQR) | 1.0 (0.4-1.5) | 1.5 (0.8-2.2) | <0.001 | 1.46 (1.23-1.74) | <0.001 |

^a^ P value was calculated with t-test, Mann-Whitney U test or Chi square test, all two-sided tests.

^b^ P value, aOR, and CI were calculated with generalized linear model with dependent variable being total estimated LR with log transformation after adjustment for age, sex, perceived stress level, post-traumatic stress disorder symptoms, short sleepers, pre-existing medical conditions, ethnicity, marital status, living alone, financial burden, COVID-19 to survey interval, and vaccination status.
